# The Muscle Activation Differences in Post-Stroke Upper Limb Flexion Synergy Based on Spinal Cord Segments: A Preliminary Proof-of-Concept Study

**DOI:** 10.3389/fneur.2021.598554

**Published:** 2021-07-22

**Authors:** Gang Liu, Chin-hsuan Chia, Wei-ning Wang, Yue Cao, Shan Tian, Xue-yan Shen, Ying Chen, Rong-rong Lu, Jun-fa Wu, Yu-lian Zhu, Yi Wu

**Affiliations:** ^1^Department of Rehabilitation Medicine, Huashan Hospital, Fudan University, Shanghai, China; ^2^Department of Rehabilitation Medicine, Ruijin Hospital, Shanghai Jiaotong University School of Medicine, Shanghai, China

**Keywords:** flexion synergy, upper limb, stroke, spinal cord segments, muscle activation

## Abstract

**Objective:** This study examined the activation difference of muscles innervated by cervical cord 5-6 (C5-C6) and cervical cord 8- thoracic cord 1 (C8-T1) in upper limb flexion synergy after stroke.

**Methods:** Surface electromyography (sEMG) signals were collected during elbow flexion in stroke patients and healthy controls. The study compared normalized activation of two pairs of muscles that could cause similar joint movement but which dominated different spinal cord segments (clavicular part of the pectoralis major, PC vs. Sternocostal part of the pectoralis major, PS; Flexor carpi radialis, FCR vs. Flexor carpi ulnaris, FCU). In each muscle pair, one muscle was innervated by the same spinal cord segment (C5-C6), dominating the elbow flexion and the other was not. The comparison of the activation of the same muscle between patients and healthy controls was undertaken after standardization based on the activation of the biceps brachii in elbow flexion.

**Results:** There was no difference between the PC and PS's normalized activation in healthy controls while the PC's normalized activation was higher than PS in stroke patients during elbow flexion. Similarly, there was no significant difference in normalized activation between FCR and FCU in healthy controls, and the same is true for stroke patients. However, the standardized activation of both FCR and FCU in stroke patients was significantly lower than that in healthy controls.

**Conclusion:** After stroke, the activation of the distal muscles of the upper limb decreased significantly regardless of the difference of spinal cord segments; while the activation of the proximal muscles innervated by the same spinal cord segment (C5-C6) dominating the elbow flexion showed higher activation during flexion synergy. The difference in muscle activation based on spinal cord segments may be the reason for the stereotyped joint movement of upper limb flexion synergy.

## Introduction

Hemiplegia is the most common sequela of a stroke and the leading cause of disability (1, 2). A considerable number of patients have chronic hemiplegia in the affected upper limb, characterized by synergistic movements (3). It is difficult to break upper limbs' synergic pattern and to induce isolated movement with practical value (4). The synergistic movements are partially voluntary yet not completely under control (5, 6). It is generally believed that decreased descending inhibitory signals or an imbalance between the inhibitory and excitatory descending signals after stroke leads to an increase in the excitability of spinal motor neurons. Overactive spinal motor neurons are sensitive to the obscured signals from the remaining pyramidal tract or extrapyramidal pathway (7), such as the reticular spinal tract (8), activating the muscles to participate in the synergistic movements (9). However, there remains an unanswered question as to why some muscles participate in synergistic movements, while other muscles are only involved to a small extent. Therefore, an investigation of the differences in the activation of different muscles in synergistic movements may help develop rehabilitation strategies to break the synergy.

In clinical practice, the typical flexion synergy of the upper limb post-stroke includes scapula lifting and internal rotation, glenohumeral joint abduction and external rotation, elbow flexion, forearm supination or pronation, and wrist and finger flexion. Interestingly, we observed that the muscles related to the flexion synergy, such as biceps, brachioradialis, and deltoid, etc., were dominated by the same spinal cord segments, mostly cervical cord 5-6 (C5-C6). Therefore, it was speculated that the motor neurons innervating the muscles that produce the upper limb flexion synergy might have a characteristic configurational relationship. In other words, the motor neurons from C5-C6 may generate synchronous excitation during an attempt to flex the elbow. According to this hypothesis, it can be inferred that the muscles that can evoke similar joint movement but are innervated by different spinal cord segments might display different muscle activation when the upper limb manifests flexion synergy. To verify the hypothesis, this study aimed to compare the activation of two pairs of muscles, one from proximal limb [clavicular part of the pectoralis major (PC) innervated by C5-C6 vs. sternocostal parts of the pectoralis major (PS) innervated by C7-T1], and one from distal limb [flexor carpi radialis (FCR) innervated by C5-C6 vs. flexor carpi ulnaris (FCU) innervated by C8-T1], during elbow flexion (elbow flexors are innervated by C5-C6).

## Methods

### Study Design

This study was designed as a case control study. It was approved by the Ethics Committee of Huashan Hospital and was registered on the Chinese Clinical Trial Registry (ChiCTR2000030178).

### Participants

Stroke patients were recruited from December 2019 to June 2020. Patients were strictly chosen according to the diagnostic criteria of the stroke (10). The inclusion criteria included: (1) age 20–75 years; (2) first-ever cerebrovascular episode; (3) Brunnstrom stages of the affected upper limb were II and III; (4) no cognitive deficit, aphasia, or psychiatric problems that might influence the proceeding of the investigation; and (5) no peripheral neuropathy or spinal cord injury. Healthy participants were also recruited to the control group. Written informed consent was obtained from all participants.

### Clinical and Demographic Measures

Age, gender, course of the disease, modified Ashworth scales (MAS), and the Brunnstrom stage of the affected upper limb was recorded or measured. All subjects were assessed by the same evaluator.

### Electromyographic Measures

**Figure 1:**
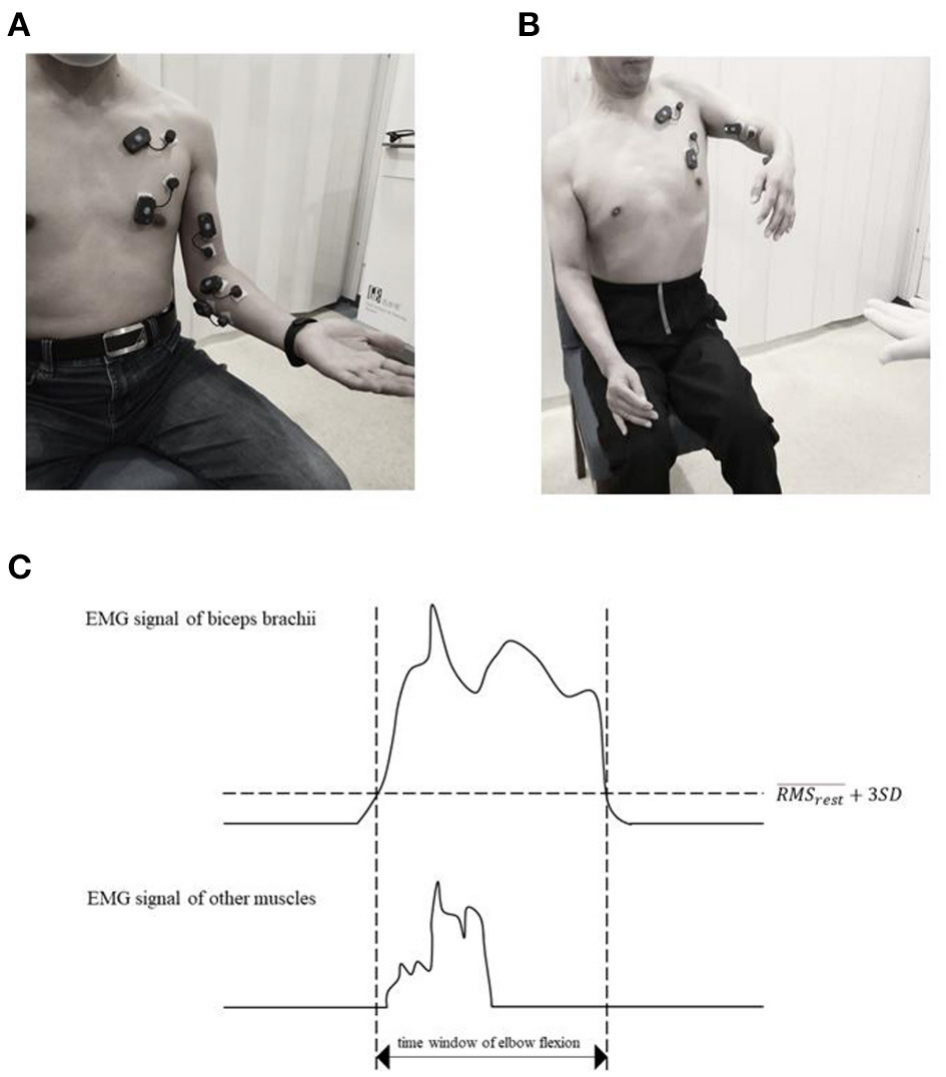
Experiment setup. The participants adopted a sitting position and were asked to flex their elbow while maintaining the neutral position of the shoulder and palm up as much as possible. **(A)** was the standard motion of the healthy control and **(B)** was the typical flexor synergy patterns of the patients with stroke. The 5 muscles EMG signals were collected. **(C)** is a diagram of selecting period of $RMS_{elbow\;flexion}$.

Before the test, the detailed process of the experiment was explained to the participants. The standard movement of elbow flexion was demonstrated. The participants were seated, with upper limbs relaxed and upper body exposed. The electrodes were placed over PC, PS, biceps brachii, FCR, and FCU, based on the Surface Electromyography for the Non-Invasive Assessment of Muscles-European Community Project (SENIAM) guideline (11) (http://seniam.org/) by the same researcher. For the muscles involved in this study but not mentioned by SENIAM, the electrode was placed on the muscle eminence along the muscle fiber direction. Surface EMG signals were recorded using BTS FREEEMG 300 with a sampling frequency of 1,000 Hz. Firstly, the baseline was recorded at rest for 15 s at least. Then, the participants were asked to flex the elbow 90°, with their palms upward (or with their best ability to perform it) and maintained at least for 5 s. Figures 1A,B illustrated the posture of healthy controls and patients following the same instruction. The maximum voluntary contraction (MVC) of the recorded muscles was obtained as follows: the participants performed full-range anti-gravity contraction of the muscle. After electrical signals were captured on the electromyogram, the participants were asked to pose the extremity in a predefined posture and performed against continuously increasing resistance applied by the researcher to the ultimate (12). Each movement was repeated three times, and the maximum value was selected as the MVC of the muscle.

### Data Analysis

The collected EMG signals were filtered under a high pass of 20 Hz and a low pass of 450 Hz, followed by rectification. The calculation method for the root-mean-square (RMS) extracted the value of a unit of 300 ms and then calculated the average value after continuous calculation.

The parameters used in the study included: (1) RMS at rest ($\bar{RMS_{rest}}$), (2) RMS of one muscle during elbow flexion ($\bar{\text{RMS}{\text{i}}_{\text{ elbow flexion}}}$), and (3) RMS of MVC (RMS_max_). The time window of all muscles was in accordance with the activation of biceps brachii, within $\bar{\text{RM}{\text{S}}_{\text{rest}}}+$3 standard deviation, was defined as the threshold of contraction (duration of contraction) (Figure 1C).

The activation of a muscle during elbow flexion was normalized by RMS_max_ of that muscle to make it comparable between different muscles. For the RMS_max_ of patients with stroke, the corresponding muscle on the unaffected side was used as a substitute due to the inability to complete the MVC in the affected extremity.

$$\begin{array}{l} \text{normalized RMS}=\left(\bar{\text{RMS}}/\text{RM}{\text{S}}_{\text{Max}}\right)*100\text{\%} \end{array}$$

To make the same muscle between patients and healthy controls comparable, we standardized the muscle activation by participant's ipsilateral biceps brachii. The Biceps was chosen because the subjects were asked to flex their elbow, and the Biceps is the active muscle that can generally reflect the degree of the cortical drive.

$$\begin{array}{l} \text{Standardized activation}=\frac{\frac{\bar{\text{RMS }{\text{i}}_{\text{elbow flexion}}}}{\text{RMS }{\text{i}}_{\max}}}{\frac{\bar{\text{RMS biceps brachi}{\text{i}}_{\text{elbow flexion}}}}{{\text{RMS biceps brachii}}_{\max}}} \end{array}$$

$$\begin{array}{l} \bar{\text{RMS }{\text{i}}_{\text{elbow flexion}}}\;:\text{average RMS of one muscle }\left(\text{eg}:\text{PC}\right)\text{during }\\ elbow\;flexion\\ \text{RMS }{\text{i}}_{\max}:\text{Maximun RMS of the same muscle}\\ \text{during MVC} \end{array}$$

### Statistical Analysis

Data were expressed as mean ± standard error. The normalized RMS of different muscles within the group were compared with the paired-sample Wilcoxon test. The standardized activation of the same muscles between healthy controls and patients with stroke was compared with the Mann–Whitney *U*-test. *P* < 0.05 indicated a statistically significant difference.

## Results

The study included 19 stroke patients (all male, average age 49 years old, duration of disease 120.7 days) and 10 health controls (both sides were tested, 20 upper limbs; all male, average age 44 years old). Detailed demographics in Table 1.

**Table 1:** Basic information of participants.

	**Patients with stroke**	**Healthy controls**
*n*	19	20
Age	48.9 ± 2.7	44.4 ± 2.6
Duration of stroke (days)	120.7	–
MAS (shoulder adduction)	1.29	–
MAS (elbow flexion)	1.61	–
MAS (wrist flexion)	1.16	–
Brunnstrom stage (upper limb)	2.79	–
Fugl-Meyer Assessment (upper limb)	11.1	–

*We use 1.5 to displace MAS Grade 1+ for ease of statistics*.

*MAS, modified Ashworth scale*.

### The Clavicular Part (C5-C6) of the Pectoralis Major Had Higher Activation Than the Sternocostal Part (C7-T1) During Elbow Flexion of the Hemiplegic Arm

**Figure 2:**
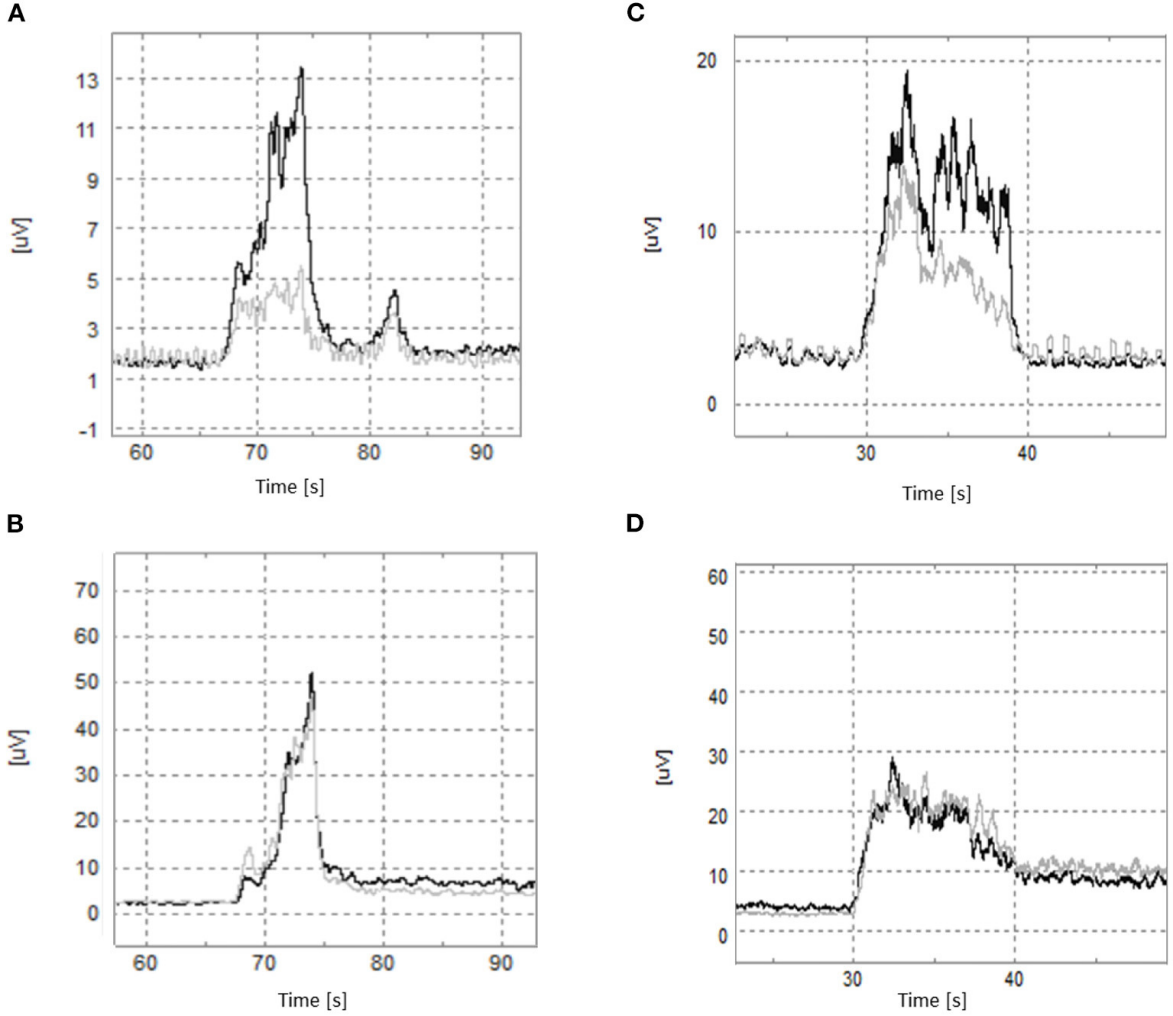
The typical RMS in elbow flexion. Patients with stroke: **(A)** the black line is the clavicle part of the pectoralis major (PC), and the gray line is the pectoralis costal part of the pectoralis major (PS). **(B)** The black line is the Flexor Carpi Radialis (FCR), and the gray line is Flexor Carpi Ulnaris (FCU). Healthy controls: **(C)** the black line is PC, and the gray line is PS. **(D)** The black line is FCR, and the gray line is FCU.

**Figure 3:**
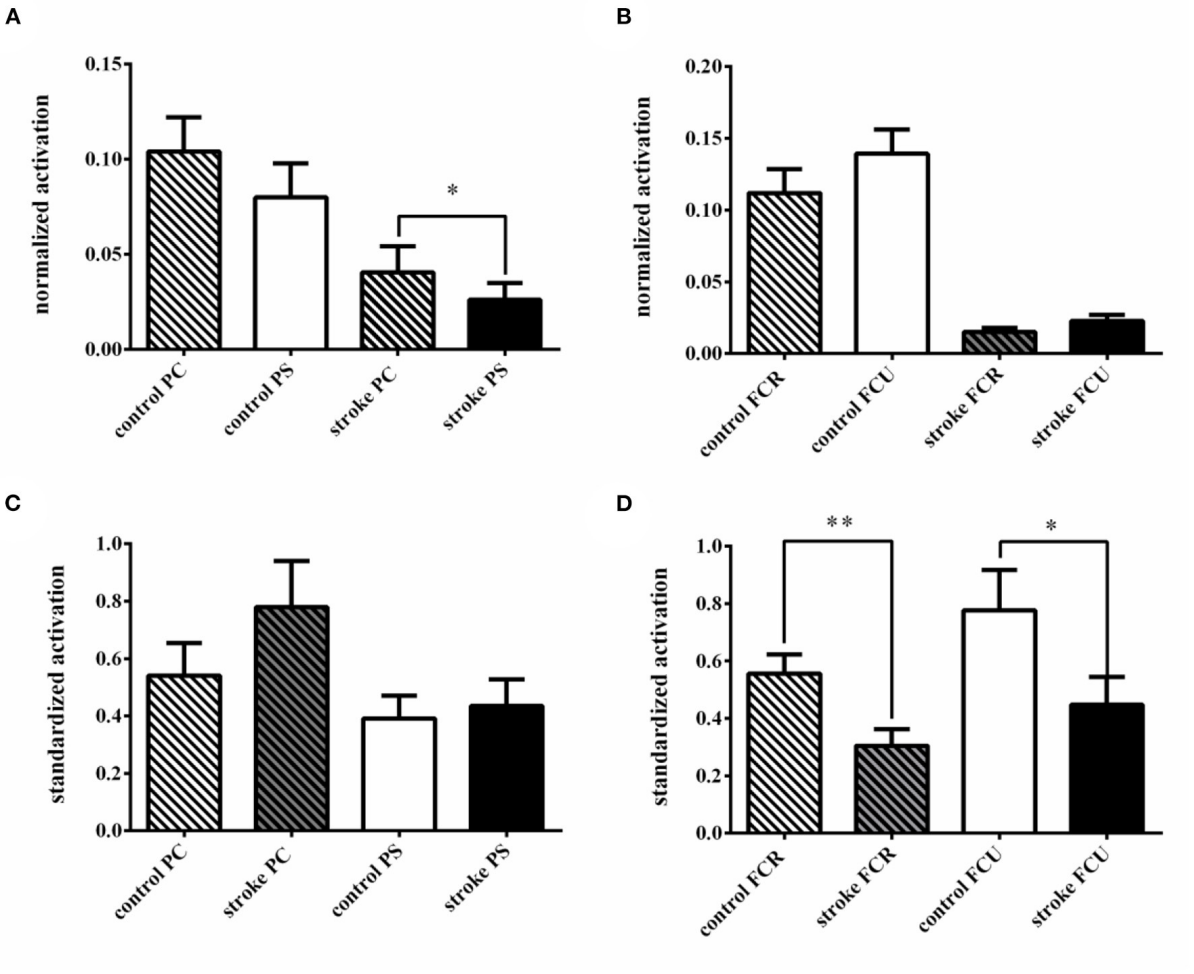
The activation difference between different spinal cord segments in elbow flexion. **(A)** Comparison of activation difference between the clavicle part of the pectoralis major (PC) and the pectoralis costal part of pectoralis major (PS) in groups; **(B)** the comparison of activation difference between the Flexor Carpi Radialis (FCR) and the Flexor Carpi Ulnaris (FCU) in groups; **(C)** comparison of the standardized activation between groups in PC and PS. **(D)** comparison of the standardized activation between groups in FCR and FCU. *p < 0.05; **p < 0.01.

No difference was found between the normalized activation of PC and PS in healthy controls during elbow flexion (PC: 0.104 ± 0.018 vs. PS: 0.080 ± 0.018; Z = −1.381, *P* = 0.167). However, the activation of PC was significantly higher than that of PS in stroke patients (PC: 0.040 ± 0.014 vs. PS: 0.026 ± 0.009; Z = −2.095, *P* = 0.036; Figures 2A,C,3A).

As for the standardized activation, no significant difference in both PC and PS was found between stroke patients and healthy controls (PC: controls 0.541 ± 0.114 vs. patients 0.779 ± 0.161; Z = −1.851, *P* = 0.064; PS: controls 0.392 ± 0.079 vs. patients 0.435 ± 0.092; Z = −0.675, *P* = 0.500; Figure 3C).

### No Spinal Cord Segment-Specific Differences in The Distal Muscle Pair of The Hemiplegic Arm

Both in healthy controls and stroke patients, there was no significant difference in normalized activation between FCR and FCU during elbow flexion (controls: FCR 0.112 ± 0.017 vs. FCU 0.139 ± 0.017; Z = −1.829, *P* = 0.067; patients: FCR 0.015 ± 0.003 vs. FCU 0.023 ± 0.004; Z = −1.587, *P* = 0.113; Figures 2B,D,3B).

The standardized activation of both FCR and FCU in stroke patients was significantly lower than healthy controls (FCR: controls 0.556 ± 0.067 vs. patients 0.305 ± 0.058; Z = −2.591, *P* = 0.010; FCU: controls 0.777 ± 0.141 vs. patients 0.447 ± 0.097; Z = −2.243, *P* = 0.025; Figure 3D).

## Discussion

The synergistic movements of the upper limb have a serious impact on the motor performance of stroke patients, which is the focus and challenge for motor recovery (3). In this study, we chose two muscle pairs that can produce the same joint movement but which are innervated by C5-C6 and C8-T1, respectively, to test our hypothesis that synchronized activation of the muscles innervated by C5-C6 results in upper limb flexion synergy. The results showed that in the patients with stroke, excitation of the spinal motor neurons innervating the proximal muscles of the upper limb appear different during flexion synergy, reflected as higher activation of the proximal muscle innervated by the same spinal cord segment dominating elbow flexion (C5-C6) than that of C8-T1. This suggested that in addition to activating the motor neurons innervating the elbow flexor muscle, the descending movement signals of elbow flexion after stroke tended to activate other adjacent motor neurons in the same spinal segment (C5-C6). However, the activation of distal muscles was significantly decreased during elbow flexion, regardless of the difference in spinal cord segments, indicating that descending motor signal after stroke was difficult to activate the neurons innervating the distal muscles.

Based on our results, the differences in muscle activation based on different spinal cord segments may account for the stereotyped pattern shown in upper limb flexion synergy. According to related basic studies, spinal interneurons might be responsible for this phenomenon. It was found that motor impulse after stroke could only activate interneurons through other descending pathways, such as the reticular spinal tract (13, 14), and simultaneously activate all motor neurons connected to the interneuron. However, it is generally believed (13, 15) that extrapyramidal descending pathways, such as the reticular spinal tract, were scarcely connected to the spinal motor neurons that innervate the distal muscles, which may be the reason why the activation of distal muscle pairs showed no difference in this study.

**Figure 4:**
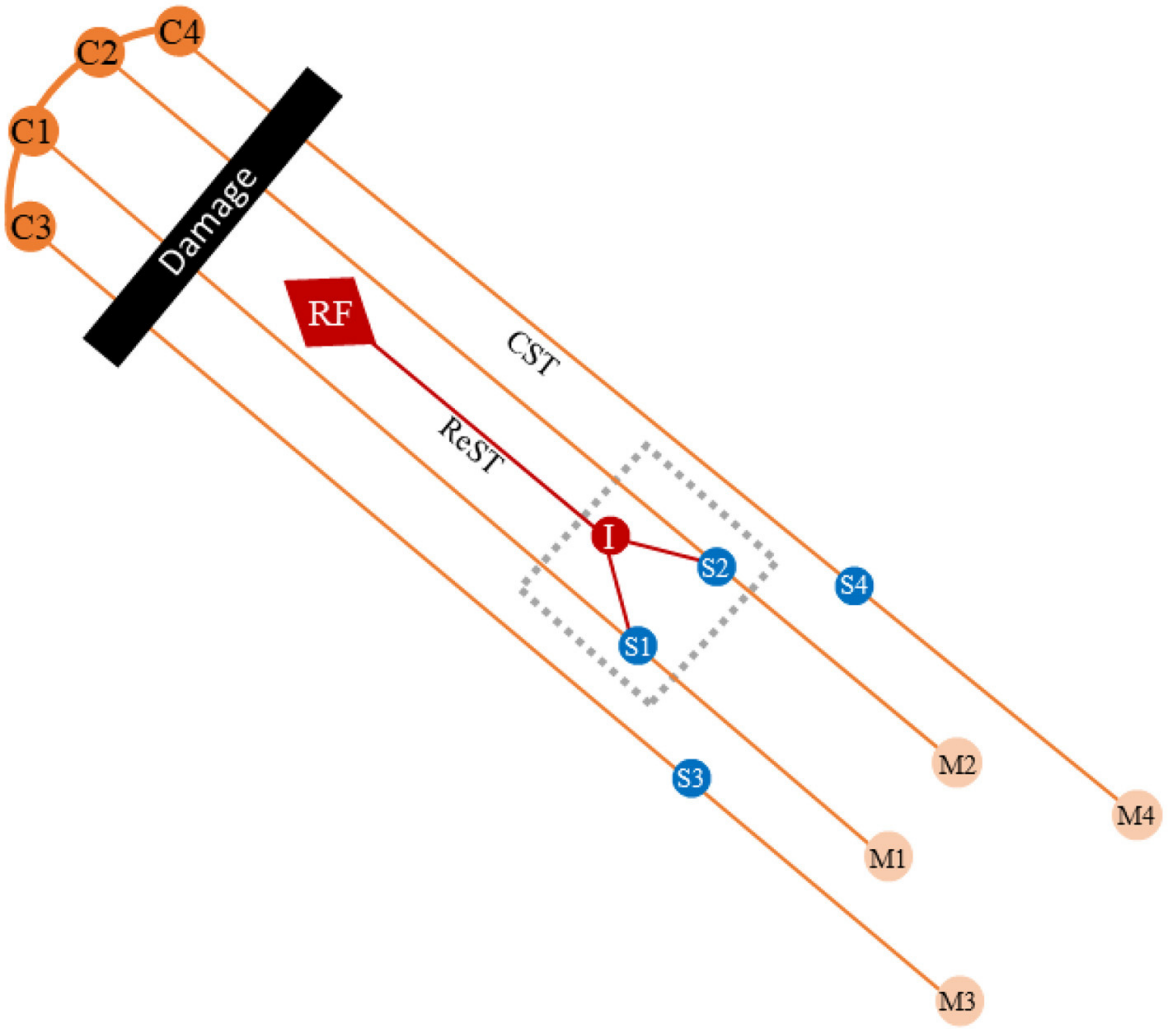
Diagram of upper limb's synergistic movements after stroke. The black bar represents the damage of the CST after stroke. The gray dotted frame represents a motor module (MM). The S1 and S2 represent the spinal motor neurons innervated proximal muscles of the upper limbs. The extrapyramidal system activates spinal motor neurons innervating the proximal muscles of the upper limb via interneurons, while the neurons innervating the distal muscles cannot be activated. C, cortex; RF, reticular formation; ReST, reticulospinal tract; CST, corticospinal tract; I, interneuron; S, spinal motor neuron; M, muscle.

Previous studies have shown that the central nervous system tended to complete motor tasks by combining limited motor modules (MMs) (5, 6, 16–18), existing at different levels (19–21) in the cortex, brainstem, and spinal cord. This has the advantage of simplifying the degree of freedom of motor control. After stroke, a loss of precise cortical control in MMs may manifest as synergistic patterns (4, 6). However, the physiological basis of MMs and the distribution of neurons belonging to the same motor module are still unclear. We speculated that spinal interneurons and their associated motor neurons together constitute motor modules. Based on our results in the proximal muscle, a possible distribution feature of the spinal motor neurons in modules related to upper limb flexion synergy after stroke, that is, anatomical proximity can be inferred (Figure 4).

However, there might be other distribution rules. It has been reported that repeated training may strengthen the connection among a specific group of motor neurons, making accordance with excitability and forming new MMs (22). Further studies are needed to learn the distribution features of the spinal motor neurons of MMs from the perspective of functional correlation.

The current study was subject to some limitations. Firstly, only the biceps were used as the elbow flexor because of the technical limitations of the sEMG electrode (e.g., when forearm pronation occurs, the electrode placed on the surface of the brachioradialis will shift on the extensor carpi muscle due to the slide of the skin). Secondly, the study set no stabilization of trunk and shoulder joint to make flexion synergy fully manifest in stroke patients, which may affect the collected EMG signals. In addition, sEMG electrodes were overlaying different muscles during movement, making the detection of the electromyographic signal of specific muscles difficult, especially FCR and FCU (interfered by the digital flexor). As for the placement of electrodes, because there is no uniform reference for some relevant muscles in this study, they were placed on the most obvious part of the muscle eminence along with the muscle fiber according to the anatomy, which may cause the deviation of the EMG signals. The findings of this study require further verification in future studies, using other techniques.

## Data Availability Statement

The raw data supporting the conclusions of this article will be made available by the authors, without undue reservation.

## Ethics Statement

The studies involving human participants were reviewed and approved by the Huashan Hospital Ethics Committee. The patients/participants provided their written informed consent to participate in this study.

## Author Contributions

GL and YW conceived and designed the projects. GL, C-hC, and W-nW conducted the experiments. GL and C-hC analyzed the raw data. GL, C-hC, and YCa edited the english. GL, C-hC, X-yS, ST, YCh, R-rL, J-fW, and Y-lZ participated in drawing and literature review. All authors contributed clarifications and guidance on the manuscript, were involved in editing the manuscript, and read and approved the final manuscript.

## Conflict of Interest

The authors declare that the research was conducted in the absence of any commercial or financial relationships that could be construed as a potential conflict of interest.

## References

[1] Gittler M Davis AM. Guidelines for adult stroke rehabilitation and recovery. JAMA. (2018) 319:820–1. 10.1001/jama.2017.22036 29486016

[2] Katan M Luft A. Global burden of stroke. Semin Neurol. (2018) 38:208–11. 10.1055/s-0038-1649503 29791947

[3] Hatem SM Saussez G della Faille M Prist V Zhang X Dispa D. Rehabilitation of motor function after stroke: a multiple systematic review focused on techniques to stimulate upper extremity recovery. Front Hum Neurosci. (2016) 10:442. 10.3389/fnhum.2016.00442 27679565 PMC5020059

[4] Israely S Leisman G Carmeli E. Neuromuscular synergies in motor control in normal and poststroke individuals. Rev Neurosci. (2018) 29:593–612. 10.1515/revneuro-2017-0058 29397390

[5] Emanuel SR Kamran I Gannon W Edgar HT. A systematic review on muscle synergies: from building blocks of motor behavior to a neurorehabilitation tool. Appl Bionics Biomech. (2018) 2018:1–15. 10.1155/2018/3615368 29849756 PMC5937559

[6] McMorland AJC Runnalls KD Byblow WD. A neuroanatornical framework for upper limb synergies after stroke. Front Hum Neurosci. (2015) 9:6. 10.3389/fnhum.2015.00082 25762917 PMC4329797

[7] Meriel O Carson I Dewald JPA. Upper extremity motor impairments and microstructural changes in bulbospinal pathways in chronic hemiparetic stroke. Front Neurol. (2017) 8:257. 10.3389/fneur.2017.00257 28659855 PMC5468392

[8] McPherson JG Chen A Ellis MD Yao J Heckman CJ Dewald JPA. Progressive recruitment of contralesional cortico-reticulospinal pathways drives motor impairment post stroke. J Physiol. (2018) 596:1211–25. 10.1113/JP274968 29457651 PMC5878212

[9] Li S Chen YT Francisco GE Zhou P Rymer WZ. A unifying pathophysiological account for post-stroke spasticity and disordered motor control. Front Neurol. (2019) 10:468. 10.3389/fneur.2019.00468 31133971 PMC6524557

[10] Sacco RL Kasner SE Broderick JP Caplan LR Connors JJ Culebras A. An updated definition of stroke for the 21st century: a statement for healthcare professionals from the American Heart Association/American Stroke Association. Stroke. (2013) 44:2064–89. 10.1161/STR.0b013e318296aeca 23652265 PMC11078537

[11] Hermens H Freriks B Merletti R Stegeman D Blok J Rau G. European recommendations for surface electromyography. Roessingh Res Dev. (1999) 8:13–54.

[12] Bohannon RW. Daniels and Worthingham's Muscle Testing: Techniques of Manual Examination. 7th ed. (Book Review). Physical therapy. Philadelphia, PA: WB Saunders Co (2003).

[13] Jang SH Chang CH Lee J Kim CS Seo JP Yeo SS. Functional role of the corticoreticular pathway in chronic stroke patients. Stroke. (2013) 44:1099–104. 10.1161/STROKEAHA.111.000269 23444306

[14] Schulz R Park E Lee J Chang WH Lee A Kim YH. Synergistic but independent: the role of corticospinal and alternate motor fibers for residual motor output after stroke. Neuroimage Clin. (2017) 15:118–24. 10.1016/j.nicl.2017.04.016 28516034 PMC5426012

[15] Baker SN. The primate reticulospinal tract, hand function and functional recovery. J Physiol London. (2011) 589:5603–12. 10.1113/jphysiol.2011.215160 21878519 PMC3249036

[16] d'Avella A Saltiel P Bizzi E. Combinations of muscle synergies in the construction of a natural motor behavior. Nat Neurosci. (2003) 6:300–8. 10.1038/nn1010 12563264

[17] Ivanenko YP Poppele RE Lacquaniti F. Five basic muscle activation patterns account for muscle activity during human locomotion. J Physiol London. (2004) 556:267–82. 10.1113/jphysiol.2003.057174 14724214 PMC1664897

[18] Zhao K Zhang Z Wen H Wang Z Wu J. Modular organization of muscle synergies to achieve movement behaviors. J Healthcare Eng. (2019) 2019:8130297. 10.1155/2019/8130297 31827741 PMC6885185

[19] Godlove J Gulati T Dichter B Chang E Ganguly K. Muscle synergies after stroke are correlated with perilesional high gamma. Ann Clin Transl Neurol. (2016) 3:956–61. 10.1002/acn3.368 28097208 PMC5224817

[20] Overduin SA d'Avella A Roh J Carmena JM Bizzi E. Representation of muscle synergies in the primate brain. J Neurosci. (2015) 35:12615–24. 10.1523/JNEUROSCI.4302-14.2015 26377453 PMC4571600

[21] Roh J Cheung VCK Bizzi E. Modules in the brain stem and spinal cord underlying motor behaviors. J Neurophysiol. (2011) 106:1363–78. 10.1152/jn.00842.2010 21653716 PMC3174810

[22] Allen JL McKay JL Sawers A Hackney ME Ting LH. Increased neuromuscular consistency in gait and balance after partnered, dance-based rehabilitation in Parkinson's disease. J Neurophysiol. (2017) 118:363–73. 10.1152/jn.00813.2016 28381488 PMC5501921

